# Experimental Investigation of Methyl Methacrylate in Stirred Batch Emulsion Reactor: AGET ATRP Approach

**DOI:** 10.3390/ma13245793

**Published:** 2020-12-18

**Authors:** Mohammed Awad, Thomas Duever, Ramdhane Dhib

**Affiliations:** Department of Chemical Engineering, Ryerson University, 350 Victoria Street, Toronto, ON M5B 2K3, Canada; mohammed.awad@ryerson.ca (M.A.); tom.duever@ryerson.ca (T.D.)

**Keywords:** AGET ATRP, ascorbic acid, batch reactor, MMA, two-step emulsion

## Abstract

This study examines the ab initio emulsion atom transfer radical polymerization (ATRP) initiated by an eco-friendly reducing agent to produce poly(methyl methacrylate) (PMMA) polymer with controlled characteristics in a 2 L stirred batch reactor. The effect of the reaction temperature, surfactant concentration, monomer to water ratio, and stirring speed was thoroughly investigated. The results showed that PMMA coagulation becomes quite severe at a certain temperature threshold. However, the coagulation could be avoided at mild reaction temperature, since the outcomes showed that loading more surfactant to the system under high mixing speed has balanced the polymer mixture and yielded high monomer conversion. The PMMA product was analyzed by gravimetry and GPC measurements and after 5 h of polymerization at a reaction temperature of 50 °C, monomer conversion of 64.1% was obtained, and PMMA polymer samples produced had an average molar mass of 4.5 kg/mol and a polydispersity index of 1.17. The structure of the PMMA polymer was successfully proved by FTIR and nuclear magnetic resonance (NMR) spectroscopy. The results confirm the living feature of MMA AGET ATRP in emulsion medium and recommend further investigation for other types of surfactant.

## 1. Introduction

All commercial polymers over the last century were produced by the free radical polymerization (FRP) technique. The main advantages of FRP over living ionic polymerization are simply the robustness concerning impurities and the FRP applicability to a variety of monomers. However, the incidence of transfer and termination reactions is a major drawback for the FRP and lead to limit the FRP fine-tuning over molecular weight distribution. Consequently, controlled radical polymerization (CRP) has emerged, which incorporates the aspects of both living ionic and conventional radical polymerizations. Past studies have demonstrated that CRP can synthesize polymers with relatively low average molecular weights, narrow molecular weight distribution (MWD), effective end group functionality, and diverse molecular architectures [1,2]. 

In particular, the atom transfer radical polymerization (ATRP) has gained a tremendous popularity in polymer chemistry in the last two decades. The salient advantages of ATRP compared to other CRP methods are the ease of operating ATRP for a wide range of monomers and over a wide range of temperatures as well as the ability of ATRP to produce high-purity block (co)polymers with end group functionality [3,4,5]. For economic and environmental reasons, conducting polymerization in emulsion media has made it more convenient for industrial settings [6,7]. An in-depth literature survey reveals that the majority of ATRP investigations have been done in solution or bulk instead of emulsion media [8]. To date, miniemulsion polymerization has been the most successful approach to perform dispersed ATRP. Even though the environment of the miniemulsion system is almost similar to bulk [9,10]. However, synthesis of various well-defined polymers has been reported using ATRP in miniemulsion systems [11,12,13]. In contrast, the major drawback of miniemulsion ATRP is the ability to upscale the application industrially because it requires to operate high shear forces. 

It is well-known that emulsion polymerization is a delicate physiochemical system since its hydrodynamic behavior is non-Newtonian and it is highly nonideal in terms of the thermodynamics equilibrium. As a result, wide particle size distribution (PSD), loss of control over the polymerization, and low initiation efficiency have emerged when carrying out ATRP in emulsion media [14]. A normal ATRP initiation scheme was used as a first attempt to extend ATRP to an emulsion system [15]. This trial took place in 1998 and was followed by many efforts [16,17,18]. All earlier attempts reported good control over molecular weight and MWD. However, the main challenge was the colloidal stability. In addition, the suspension system was captured on the product more than the emulsion system. Matyjaszewski et al. [19] reported poor colloidal stability when normal ATRP in the emulsion was conducted using a hydrophilic initiator. In our case, Makino et al. [20] were the first to report the ATRP of methyl methacrylate (MMA) in an aqueous medium. A PMMA polymer with a relatively narrow MWD of 1.63 was obtained at a high monomer conversion of 80–90%. The next level was to use the reverse ATRP initiation technique to approach a successful emulsion ATRP and to ensure no monomer droplets nucleation occurred [21,22]. In fact, the reverse ATRP has overcome the colloidal stability because the nucleation and initiation process was very identical to the conventional radical emulsion polymerization. However, it requires the use of a high amount of undesired catalyst compared to other ATRP initiation schemes. Therefore, Min et al. [23,24] attempted to apply the relatively new initiation technique of activators generated by electron transfer (AGET) ATRP, which incorporated environmentally friendly reducing agents and requires way fewer amounts of catalyst complex compared with reverse ATRP. The AGET ATRP technique was performed in the emulsion medium because of the stable and oxidative copper (Cu^II^) complex that is inherently incorporated in the initiation step. Besides, the amount of the reducing agent, which plays a major role in controlling the polymerization rate, can be readily adjusted. Furthermore, the reducing agents employed in AGET ATRP likely consume oxygen traces in the reaction medium and, consequently, help bypass the deoxygenation stage. It is important to highlight that, according to previous studies [25,26,27], reducing agents are responsible for converting the deactivator (Cu^II^) into an activator (Cu^I^) of the catalyst. 

In emulsion ATRP, the way in which the hydrophobic catalyst complex and initiator are compartmentalized inside the monomer droplets, and their postulated mass transfer into the growing polymer particles, can significantly affect monomer droplet nucleation. For a relatively high solid content or a larger polymer particle size, colloidal instability can become a major problem, hindering the reaction progress especially at high reaction temperature and as we discussed earlier. This particular problem can be alleviated by performing a seeded or microemulsion, followed by regular emulsion polymerization. The two-step method adopted in this study involved the addition of a fresh monomer during the reaction. This stepwise procedure allows researchers to sustain the characteristics of living controlled polymerization, which depends strongly on maintaining a reasonable amount of the monomer in a dispersed reacting system. In addition, the ligand must be highly soluble in the organic phase. Polymer particles are first nucleated during the microemulsion stage, and more monomer is subsequently added into the ongoing reaction to sustain a reasonable behavior of emulsion polymerization [24]. Another postulated feature of the two-step procedure is a possible enhancement in the mass transfer of the initiator and catalyst complex into monomer particles. Without the two-step procedure, they could remain trapped inside the polymerizing particles (i.e., nucleated micelles) during the microemulsion stage.

Based on literature survey, many previous studies [28,29,30,31,32,33] reported a well-controlled AGET ATRP of MMA in emulsion polymerization using the two-step method to synthesize polymers with a low polydispersity index (Ð), narrow experimental number-average molar mass (M_n_), and high initiation efficiency. For instance, Cheng et al. [34] performed a well-controlled emulsion AGET ATRP of MMA using a surface-active initiator. Li [35] conducted a two-step AGET ATRP of MMA and good colloidal stability for both microemulsion and emulsion polymerization was reported. Wei et al. [36] applied the copper capture agent as a surfactant-ligand design with a catalyst complex to carry out an emulsion ATRP of MMA. The authors maintained an acceptable control over the polymerization while reducing the loss of catalyst complex and the surfactant concentration in the aqueous phase. Li et al. [37] investigated a two-step system via AGET ATRP in the emulsion medium to prepare PMMA macromonomers for poly(n-butyl acrylate)-g-poly(methyl methacrylate) (PnBA-g-PMMA) graft copolymerization. 

It is important to highlight that an in-depth understanding of the chemistry of ATRP in emulsion systems and the related aspects of chemical kinetics for such a system are not well documented in the literature since ATRP is still largely unexplored in dispersed systems [36]. In fact, there is a lack of detailed experimental data to cover a range of reaction conditions wide enough for a better understanding of the ATRP kinetics and hence, to develop ATRP mechanistic models in emulsion media. In addition, most experimental investigations have been done in small laboratory-scale reactors (5–10 mL ampoules) in which stirring and the miscibility of the reacting medium were not considered. Recently, Lorandi et al. [38] reported the AGET ATRP of poly(meth)acrylates using an ab initio emulsion with a 100 mL reaction volume. A well-controlled (co)polymer was produced and the researcher, therefore, suggested a facile scale-up of this technique. Our research group studied the AGET ATRP of MMA in a 2 L batch reactor using a two-step procedure, and successful studies were reported in such a reactor vessel. The previous studies revealed that the amount of stirring and nitrogen purging have a considerable impact on the reaction, while obtaining high monomer conversion was a challenge [39,40]. 

The present study further investigated the effects of the reaction temperature, the surfactant concentration, the amount of water added to the system, and the stirring speed on the performance of a 2 L batch reactor under different reaction conditions. The focus of this study is to explore the colloidal stability of emulsion AGET ATRP using a two-step polymerization technique in order to enhance the monomer conversion rate for more PMMA product without polymer coagulation. 

## 2. Experimental Procedure

A detailed description of the experimental setup is reported in our previous study [41]. However, a brief explanation was provided in the following sections to explain the experimental synthesis and tests that have been carried out in this study. 

### 2.1. Polymerization Reactor 

The reaction was carried out in 2 L stainless steel reactor (PARR, model 4530, Parr Instrument Company, Moline, IL, USA). The reactor vessel is connected with a pressure gauge and a heating/cooling bath (Cole-Parmer Polystat H28L, Cole-Parmer, Montreal, QC, Canada) operated by a U-shaped cooling coil. In addition, the reactor has a temperature control unit (model 4848, Parr Instrument Company, Moline, IL, USA) connected to a thermocouple type J to measure the interim reactor temperature and equipped with an impeller speed motor and controller. In addition, the reactor vessel is mounted with an inlet and outlet ports used for chemicals injection, samples collecting, and gas purging. A dummy run is recommended prior the experimental trial in order to check the accuracy of the heater control, the motor speed, and the reactor pressure and temperature. The autotuning procedure is usually carried out with distilled water for about an hour. 

### 2.2. Materials

All the reactants involved in this study were purchased from Sigma Aldrich (Oakville, ON, Canada) with the exemption to the oxidative catalyst copper bromide (CuBr_2_, 99%, VWR, Mississauga, ON, Canada) and the initiator ethyl-2-bromoisobutyrate (EBiB, >98%, VWR, Mississauga, ON, Canada). The compounds of nonionic surfactant polyoxyethylene (20) oleyl ether (Brij 98), the hydrophobic ligand 4,4′-Dinonyl-2,2′-dipyridyl (dNbpy, 97%), and the benign reducing agent L-ascorbic acid (C_6_H_8_O_6_, 99%) were used as received. The monomer methyl methacrylate (MMA, 99%) was purified by passing through a column packed with an inhibitor remover, and then, the MMA was placed in a dark jug and stored in the refrigerator under a temperature between 0 and 2 °C. The emulsion medium in all cases was the distilled water. Standard polystyrene (PS) samples were purchased from PolyAnalytik (London, ON, Canada) and was used as received. The polymer samples were precipitated by methanol (99.8%, VWR, Mississauga, ON, Canada). 

### 2.3. Two-Step Emulsion Polymerization

Table 1 summarizes the experimental conditions of the first and second experimental sets. The first experimental set (E1 and E2 runs) was carried out for 5 h reaction time for the E2 trial, and a reaction time of 3 h for the E1 trial, due to coagulation. The reaction recipe amounts were taken from a previous study using a 2 L reactor [39]. The second experimental set (E3 to E13 trials) was carried out for 5 h reaction time for trials E4 to E13 and 3 h for the E3 trial, again, due to coagulation. The reaction recipe amounts for the E4 trial were taken from our previous study, but with a different ligand [41]. The two-step experimental method was used in both experimental sets, but with different amounts of reactants. In addition, both experimental sets were conducted under a constant 20 psig nitrogen pressure. The amount of reactants used in the E1 and E2 trials was identical. For the E4 trial, a lower catalyst complex amount was used along with higher amounts of the surfactant, initiator, and reducing agent. The second experimental set was carried out to examine the colloidal stability of the improved monomer conversion system. All runs used the same amount of reactants for the E4 trial but one factor was changed at a time. For instance, the E3 and E5 runs had the same amount of reactants as the E4 trial, but the reaction temperature was set to 60 and 40 °C, respectively. Moreover, trials E6 to E8 had the same amount of reactants as the E4 trial, but the amount of Brij 98 used was 5, 10, and 15 g, respectively. The same procedure was followed for the rest of the experimental cases, which all had the same amount of reactants as the E4 trial, but the amount of water was 400, 450, and 500 mL for E9 to E11 trials while the stirring speed was 100 and 150 rpm for E12 and E13 trials.

We need to point out that the required amount of MMA monomer and AA reducing agent were splitted into two portions: MMA(I) and MMA(II) and AA(I) and AA(II), respectively. All experimental trials have a fixed amount of MMA monomer and equals 56.16 g (MMA(I) = 14.04 g and MMA(II) = 42.12 g). The two-step procedure is characterized by two significant reaction mediums: the transparent microemulsion medium followed by emulsion medium. In the beginning, the organic phase consists of the catalyst complex (CuBr_2_/dNbpy) and EBiB dissolved in the MMA(I) under continuous stirring to form homogeneous organic solution. Meanwhile, the Brij 98 surfactant was dissolved under stirring in the distilled water to form the aqueous solution. The organic solution was poured slowly into the aqueous solution under stirring to form transparent greenish-blue microemulsion. For simplicity purposes, the mixing process of the organic and aqueous solutions were conducted in the presence of air for both experimental sets. The whole transparent microemulsion mixture was then transferred into the reactor and purged with nitrogen. The first experimental set was purged with nitrogen for 10 times, while the second experimental set was purged with nitrogen for 6 times only. The reason behind minimizing the number of the nitrogen purging was to avoid losing large amounts of EBiB because it is a highly volatile material. Later on, another aqueous solution was prepared that contained the total amount of AA dissolved in 20 mL of distilled water. After the reactor reached the desired reaction temperature, the AA(I) dissolved in 10 mL distilled water was injected into the reactor to activate the catalyst and start the polymerization. After some time of initiation (12 min for the first experimental set and 15 min for the second experimental set), the MMA(II) and the AA(II) were injected to the ongoing microemulsion polymerization to form a milky emulsion polymerization. The PMMA polymer samples were withdrawn at specific times during the polymerization reaction and then immediately shaken and placed in the refrigerator to quench the reaction.

### 2.4. Characterization

The monomer conversion was calculated by gravimetry procedure. The PMMA samples were collected at desired times and weighed in a known bulk aluminum cup and placed in a vacuum oven (VWR, 550 watts, Mississauga, ON, Canada) at 50 °C for 24 h. Afterward, the dried samples were weighed again to calculate the polymer content. Gel permeability chromatography (GPC, Viscotek TDA model 302, Houston, TX, USA) utilized the polystyrene standards calibration to determine the experimental M_n_ and Ð of the PMMA product samples. This GPC model has a triple detectors. In addition, the tetrahydrofuran (THF) was used as the mobile phase in the GPC column. The temperature was maintained at 22 °C for both the GPC detectors and column. For GPC measurements, the dried polymer samples were dissolved in the THF and passed through a filter packed with aluminum oxide to remove the residual CuBr_2_. Microtrac S3500 equipment (NIKKISO Group, Hebron, KY, USA) was used to determine the average size of the PMMA polymer particles based on the laser diffraction rule. Moreover, the Fourier-transform infrared (FTIR, Cary 630 Spectrometer, Agilent Technologies, Mississauga, ON, Canada) spectroscopy equipped with a 360° swivel press was used to confirm that the PMMA polymer was produced. Besides, the nuclear magnetic resonance spectroscopy (NMR, Bruker 400 MHz with AVII module, Milton, ON, Canada) with a multiprobe was utilized to define the structure of the PMMA polymer samples.

### 2.5. Measurement Reliability and Replication

The monomer conversion for each experimental run was repeated two times and a one-time repeat of GPC analysis for each sample collected in all cases. In addition, fully independent replicates were performed for runs E1 to E13. The values were plotted in the successive figures to prove the reproducibility of the data. In this study, the values of Ð and M_n_ of the replicated trials were reported at 1 and 3 h only for both experimental sets. However, for the first experimental set, monomer conversion of PMMA samples were analyzed in series at 0.5 and 3 h for E1_Replicate and at 1 and 3 h for E2_Replicate. 

## 3. Results and Discussion

**Figure 1:**
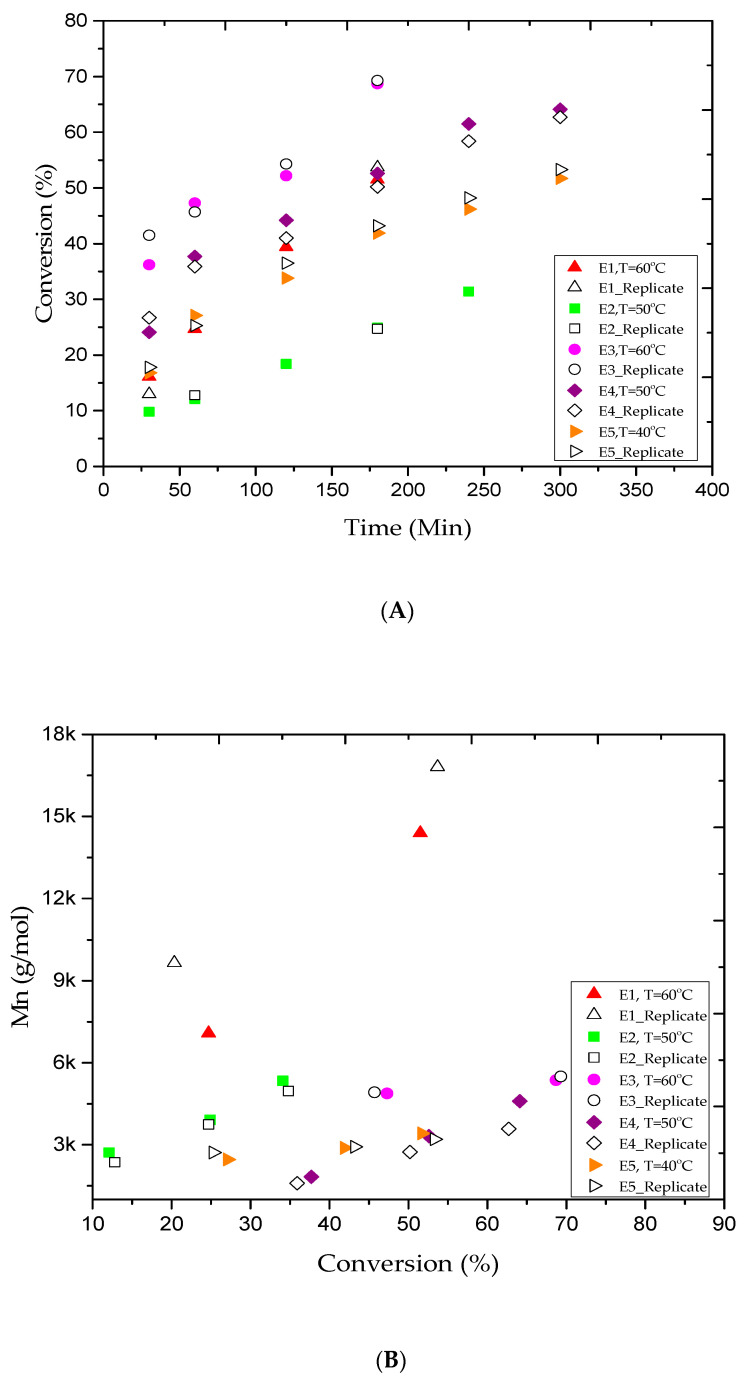

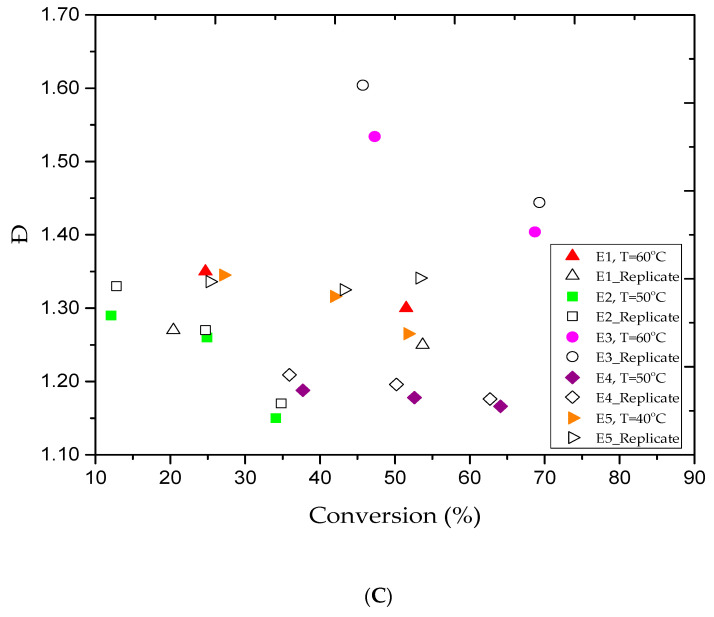
For experiments E1–E5: (**A**) methyl methacrylate (MMA) conversion versus reaction time, (**B**) variations of experimental number-average molar mass (M_n_) versus methyl methacrylate (MMA) conversions, and (**C**) variations of the polydispersity index (Ð) versus methyl methacrylate (MMA) conversions.

As shown in Table 1, 13 experimental trials were conducted under different conditions to produce PMMA polymer. Figure 1A shows a conversion of 51.5% in experiment E1 (Table 2) at 3 h of reaction time. The experimental data showed a rapid increase in the monomer conversion, which led to instability and caused the solution to coagulate. Consequently, trial E2 was conducted at a lower temperature of 50 °C while using the same reaction recipe in E1 in order to suppress the formation of polymer aggregation. The monomer conversion progressed steadily to 24.9% in 3 h. The reaction was left to proceed for more 2 h, but the final conversion only reached 34.1%, and no polymer coagulation was observed. In fact, lowering the reaction temperature has contributed to achieve better latex stability. The living characteristics of AGET ATRP polymerization are commonly defined by producing a polymer with a narrow polydispersity index (Ð = M_w_/M_n_) and low molecular weights [42]. In this study, these features were observed in all experimental trials, as shown in Table 2. For example, the polymer samples collected in experiments E1 and E2 after 3 h of reaction time had a narrow Ð values of 1.30 and 1.26, respectively. Besides, the polymer samples had relatively low M_n_ of 14.3 kg/mol in E1 and 3.9 kg/mol in E2. The Ð and M_n_ trends for both experiments are shown in Figure 1B,C, respectively. A decrease in Ð for high conversion is a typical feature of the reversible deactivation radical polymerization (RDRP). The results confirm that the reaction temperature has a fundamental effect on the PMMA polymer samples produced through AGET ATRP emulsion. However, at high conversion rates, the latex stability of the polymer product remains a challenging issue. The occurrence of latex instability is likely due to a large number of oligomeric chains that were unable to grow rapidly enough in order to effectively stabilize the polymer particles. In other words, with the increase in MMA conversion, the transfer of the catalyst (CuBr_2_) species into the aqueous phase led to reduce the Brij 98 solubility in water, and therefore, contributes to impede the polymer particles stabilization [24]. In fact, to limit the chances of coagulation occurrence, a restricted protocol needs to be followed during each reactant selection to overcome the aggregation constraints at high monomer conversion. For example, it is believed that for stable and successful emulsion AGET ATRP system, a high hydrophobic ligand needs to be involved when a nonionic surfactant is used, while an anionic surfactant is quite good with high hydrophilic ligand. On the other hand, it is well-known that high reaction temperature increases the rates of polymerization and equilibrium reaction in ATRP system, and hence, it further improves monomer conversion [43]. Moreover, the concentration of both the activator and the deactivator in the catalyst complex is carefully adjusted in the ATRP equilibrium reaction to obtain better monomer conversion [44]. Thus, a balanced and continuous rate of exchange between the deactivator and activator can enhance the ATRP equilibrium reaction rate and balance the CuBr/CuBr_2_ ratio. In addition, a large amount of surfactant can generate more micelles for nucleation and lead to obtain more polymer content. Furthermore, the initiator is an important reactant that should be kept at a reasonable level during the ATRP polymerization. Since EBiB is very volatile and can easily be carried away during the purging period, more EBiB had to be added to compensate for any loss and ensure that enough of the initiator remained inside the reactor vessel to sustain the reaction [41]. In addition, variation in the initiator to monomer ratio can also affect the molecular weight. It has been reported that increasing the initiator concentration lowers the polymer molecular weights [5,15,41,45].

The experimental conditions used in the E1 and E2 trials did not result a high monomer conversion rate, even at 60 °C. For this reason, three more tests (E3, E4, and E5) were conducted using the experimental conditions listed in Table 1, and the results are summarized in Table 2. Figure 1A shows the MMA conversion versus time for the E3, E4, and E5 trials. MMA conversion of approximately 68% was yielded after 3 h of reaction time at 60 °C. The reaction was once again discontinued due to the occurrence of coagulation. Therefore, the E4 and E5 trials were performed in series at lower temperatures of 50 and 40 °C to prevent polymer aggregation and allow the reaction to proceed further to higher conversion rates. At E4 and E5 trials, the polymer content increased gradually to approximately 53% and 42% after 3 h and reached nearly 64% and 52% after 5 h, respectively. In both trials, milky homogenous polymer samples were collected, and polymer coagulation was not an issue. The results confirmed the livingness of the PMMA polymer produced using emulsion two-step AGET ATRP in 2 L batch reactor. In fact, we felt it is crucial to analyze the results obtained in the E2 and E4 trials. In addition, according to a thorough statistical analysis study conducted by Upadhayay Regmi et al. [40], it is found that the MMA conversion remains almost invariant with EBiB variations, while the PMMA molecular weight is significantly affected. Therefore, we believe that the conversion improvement recorded in the E4 trial is likely due to the increase in the surfactant concentration and not the initiator concentration. In other words, increasing EBiB from 0.4988 (E2) to 0.6147 g (E4) did not affect MMA conversion, but contributed to lower the M_n_ of the PMMA. Consequently, we conclude that the addition of more surfactant helped to establish better latex stability and allowed the reaction to attain a higher monomer conversion rate. In addition, it is clear that the AGET ATRP of MMA becomes extremely sensitive to thermal effects as the temperature is raised to the vicinity of 60 °C while the amount of surfactant remains low. For the E3, E4, and E5 experiments, the data plotted in Figure 1B,C illustrate the M_n_ variations and the Ð values in series versus the monomer conversion rates. For the E3 trial, a broad MWD of 1.40 was obtained and the emulsion polymerization lost its stability, as well as the reaction was discontinued after 3 h of reaction as a result of coagulation. On the other hand, samples of the E4 trial were collected after 1, 3, and 5 h of reaction time for GPC analysis. Table 2 shows that low M_n_ values of 1.8, 3.3, and 4.5 kg/mol and narrow Ð values of 1.19, 1.18, and 1.17 were obtained, respectively. The results confirm the fact that the reaction temperature had a significant effect on the characteristics of the polymer product and increases the polymer content. Interestingly, and based on obtained E4 trial results, we may conclude that loading more surfactant and reducing agent will lead to increase the monomer conversion rate at lower temperatures. Therefore, it is possible to avoid the coagulation when the proper reaction conditions are carefully selected. The results confirmed that well-controlled AGET ATRP polymerization systems were obtained for the E3, E4, and E5 trials.

The significant results obtained from the E4 trial motivated us to study other effects in this particular polymerization system and under the same experimental conditions. Therefore, additional runs were conducted to investigate the colloidal stability of the MMA in this system, and as shown in Table 1. For instance, the E6, E7, and E8 trials were performed to investigate the effect of changes in the amount of surfactant while the E9, E10, and E11 trials studied the effect of the ratio of the monomer to the amount of distilled water added to the system, and finally, the E12 and E13 trials investigated the effect of the stirring speed. It is important to point out that one experimental factor was changed at a time. All other experimental conditions remained identical to the E4 trial.

**Figure 2:**
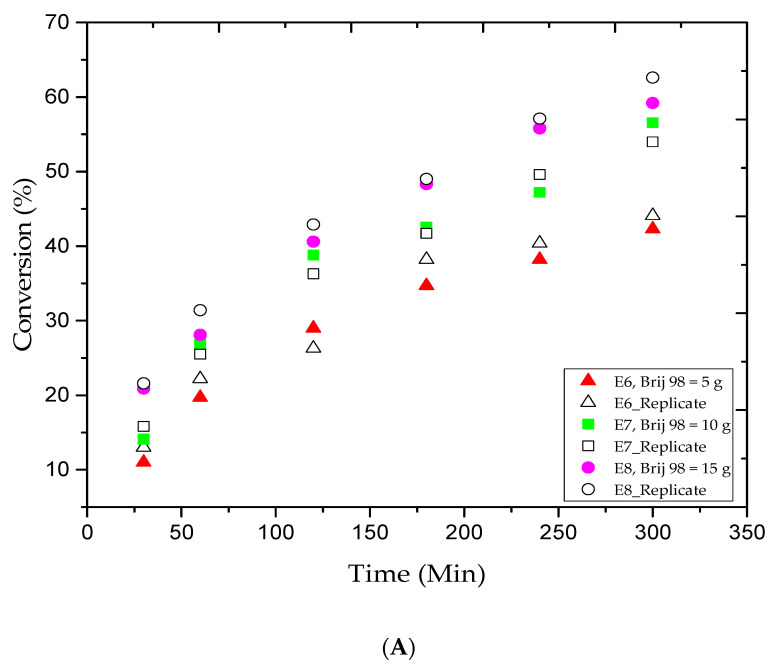

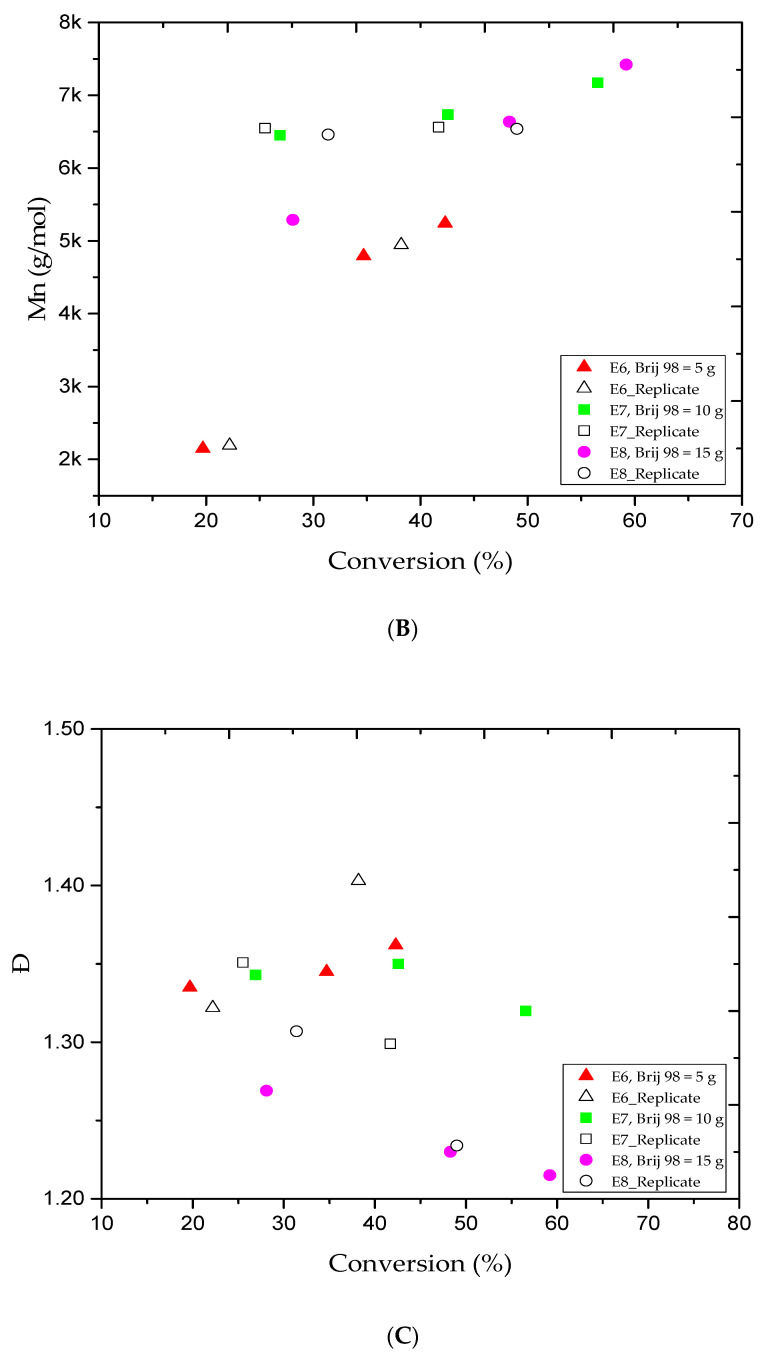
For experiments E6–E8: (**A**) methyl methacrylate (MMA) conversion versus reaction time, (**B**) variations in the experimental number-average molar mass (M_n_) versus methyl methacrylate (MMA) conversions, and (**C**) variations in the polydispersity index (Ð) versus methyl methacrylate (MMA) conversions.

The selection of surfactants in AGET ATRP emulsion is crucial. A suitable amount of surfactant is required to stabilize the latex, but the particular surfactants must not react with the other species in the medium. Figure 2A shows the MMA conversion versus reaction time for the E6, E7, and E8 trials. The plotted data indicate that the addition of more Brij 98 (from 5 to 10 and 15 g) will boost the MMA conversion rate. As a result, from 1 to 5 h of reaction time, the MMA conversion rate progressively increased from 19.7% to 42.3%, 26.9% to 56.6%, and 28.1% to 59.2%, respectively, as listed in Table 2. The results were in agreement with our outcomes in earlier trials, which confirms that additional surfactant causes more micelles to nucleate and produces more PMMA. Figure 2B,C illustrate the M_n_ and Ð variations versus the MMA conversion rate for the E6, E7, and E8 trials. The data collected by GPC analysis indicated a controlled ATRP polymerization since the average molar mass of the PMMA products increased along with the MMA conversion rate. For example, Table 2 shows that for the 1, 3, and 5 h reaction times, the M_n_ values obtained were low and gradually increased from 2.1 to 5.2, 6.4 to 7.1, and 5.2 to 7.4 kg/mol, respectively. Moreover, the E6 and E7 runs showed a slight lack of consistency, as the Ð value slightly increased and then narrowed, while in E8 trial, the Ð value consistently decreased as more MMA was converted. Based on the results obtained, one may say that the latex stability is one of the major challenges in emulsion AGET ATRP, and it is highly affected by the surfactant type and amount.

**Figure 3:**
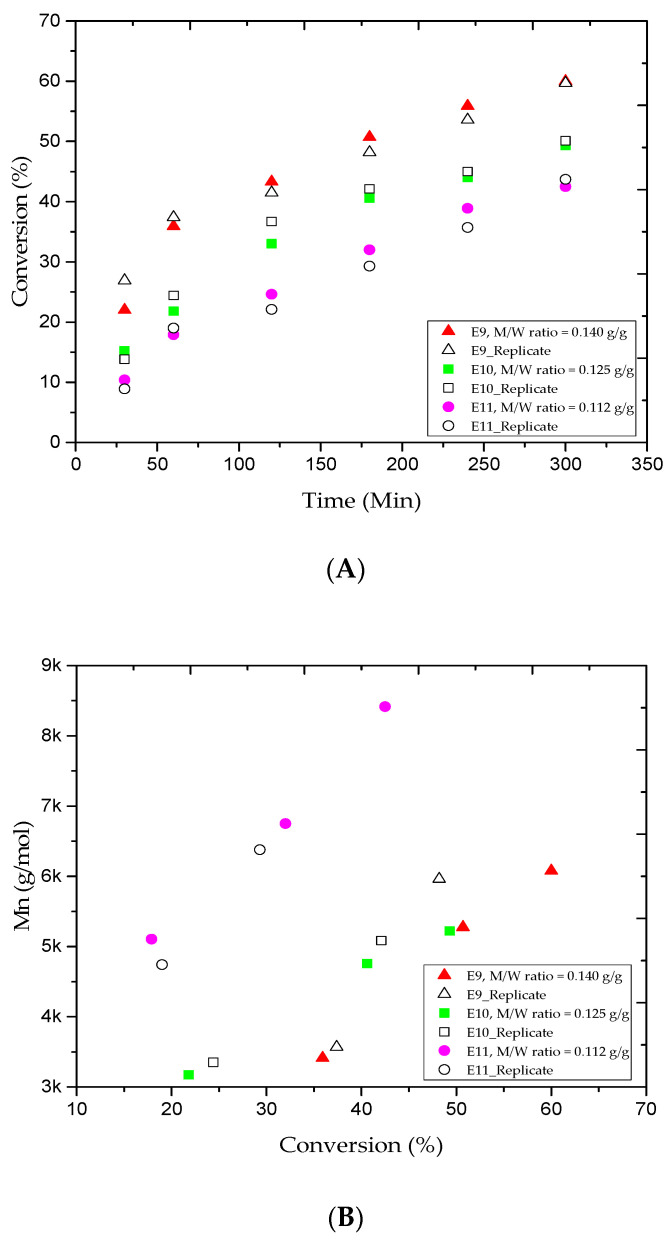

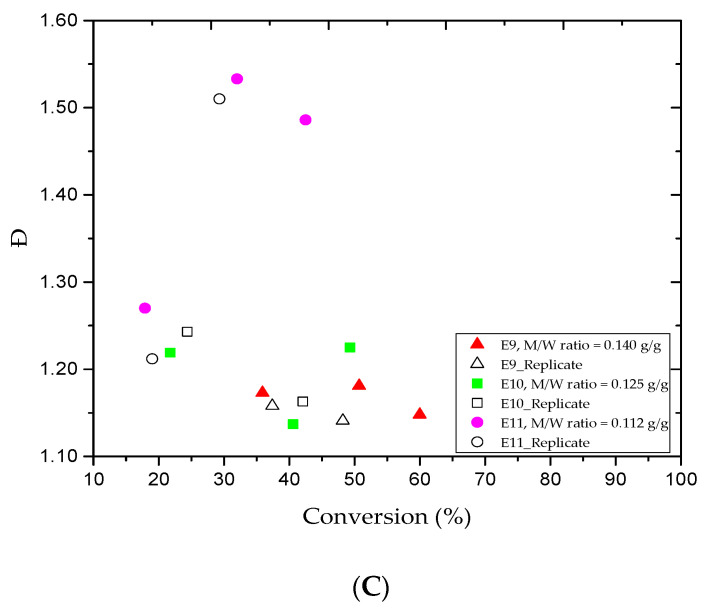
For experiments E9–E11: (**A**) methyl methacrylate (MMA) conversion rate versus reaction time, (**B**) variations in the experimental number-average molar mass (M_n_) versus methyl methacrylate (MMA) conversions, and (**C**) variations in the polydispersity index (Ð) versus methyl methacrylate (MMA) conversions.

A few studies [46,47] have looked at the effect of the amount of water on emulsion polymerization. This investigation was mainly focused on the monomer/water weight ratio (M/W). The M/W values for the E4, E9, E10, and E11 trials were 0.160, 0.140, 0.125, and 0.112 g/g, respectively. Figure 3A shows the MMA fractional conversion versus the reaction time for the E9, E10, and E11 trials. The results revealed that the addition of more water to the system led to decrease the MMA conversion. For instance, when the water amount in E9 was 400 mL, the MMA conversion rate gradually increased from 35.9% to 60.0% after the 1 and 5 h reaction periods. Besides, Table 2 tells that the results obtained were lower than those for the E4 run in which the water amount was 350 mL. Interestingly, lower MMA conversion rates were obtained at the same reaction time for the E10 and E11 trials (the water amounts were 450 and 500 mL, respectively). It is clear that a lower amount of monomer is nucleated when more water is added to the system [46]. Figure 3B,C plots the variations of the M_n_ and Ð values versus the MMA conversion rate for the E9, E10, and E11 trials. For the samples collected at the 1 and 5 h reaction times, the M_n_ values increased from 3.4 to 6.0, 3.1 to 5.2, and 5.1 to 8.4 kg/mol, respectively, as also shown in Table 2. We believe that the partitioning effect was the main reason behind the broadness of the experimental M_n_ values for E11 trial where most MMA and CuBr_2_ amounts transferred to the aqueous phase in emulsion AGET ATRP. For the same reaction period, Table 2 shows that the Ð values for the E10 and E11 trials increased rapidly from 1.22 to 1.23 and 1.27 to 1.49. This may be attributed to the fact that most of the CuBr_2_ escapes to the aqueous phase. Besides, the water-soluble reducing agent, which initiates the AGET ATRP mechanism, may require optimization.

**Figure 4:**
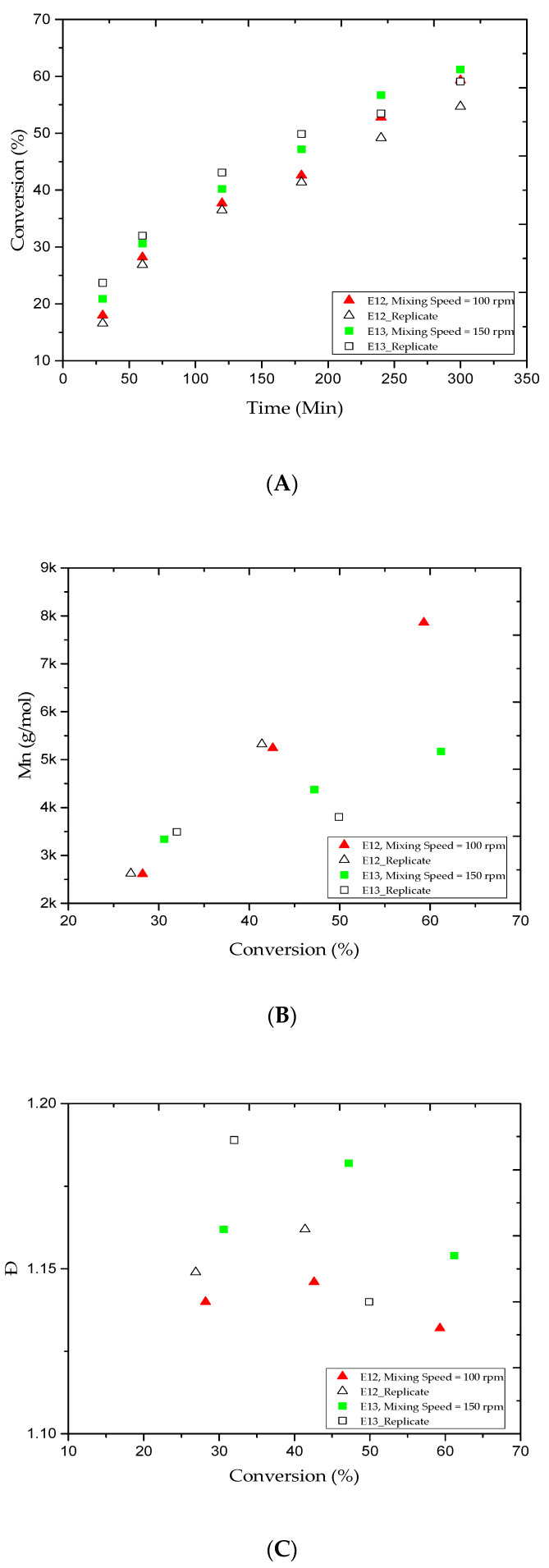
For experiments E12 and E13: (**A**) methyl methacrylate (MMA) conversion rate versus reaction time; (**B**) variations in the experimental number-average molar mass (M_n_) versus the methyl methacrylate (MMA) conversion rates; and (**C**) variations in the polydispersity index (Ð) versus the methyl methacrylate (MMA) conversion rates.

Even though, significant advances have been achieved in emulsion polymerization, the fundamental concept of mixing, which is the basis for the formation of an emulsion mixture, has not been fully delineated [48,49,50]. Therefore, the impact of stirring speed on the MMA conversion rate and average MWD was examined in this study. The polymerization process was carried out at a constant stirring speed throughout the reaction. Figure 4A shows the gradual increase in the MMA conversion versus reaction time for the E12 and E13 trials. In our case, when the stirring speed increased from 100 to 150 rpm, the polymer content increased from 28.2% to 59.3% and 30.6% to 61.2% for the 1 and 5 h reaction times, respectively. At lower stirring speed, the trials had a longer induction time before the MMA conversion rate increased [51]. Figure 4B,C shows the variations in the M_n_ and Ð values of the samples collected from the E12 and E13 trials and during the same reaction period. According to Table 2, the M_n_ values increased from 2.6 to 7.8 and 3.3 to 5.1 kg/mol, while the Ð value decreased from 1.14 to 1.13 and 1.16 to 1.15, respectively. Consequently, the good emulsification and recirculation in the E4 trial has led to observe better MMA conversion rate, M_n_, and Ð compared with E12 and E13 trials. In other words, we conclude that increasing the stirring speed has enhanced the homogeneity of emulsion AGET ATRP [52].

**Figure 5:**
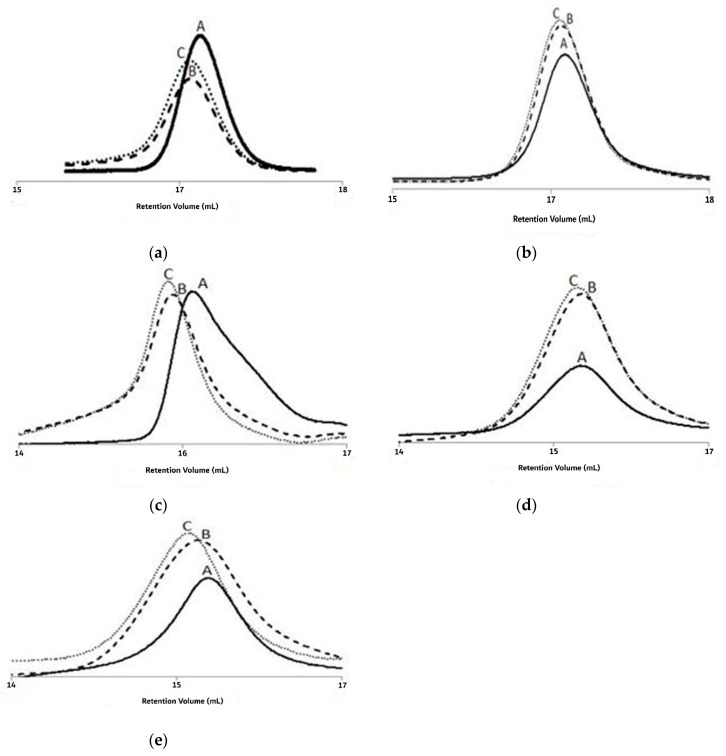
For experiments E2, E4, E6, E9, and E12: gel permeability chromatography (GPC) traces (**A**–**C**) of the poly(methyl methacrylate) (PMMA) molecular weight distribution (MWD) at 1, 3, and 5 h of reaction time. (**a**) The GPC peaks shift of PMMA for 12.1, 24.9 and 34.1% conversions, respectively in trial E2; (**b**) The GPC peaks shift of PMMA for 37.7, 52.6 and 64.1% conversions, respectively in trial E4; (**c**) The GPC peaks shift of PMMA for 19.7, 34.7 and 42.3% conversions, respectively in trial E6; (**d**) The GPC peaks shift of PMMA for 35.9, 50.7 and 60.0% conversions, respectively in trial E9; (**e**) The GPC peaks shift of PMMA for 28.2, 42.6 and 59.3% conversions, respectively in trial E12.

Figure 5 shows the GPC traces of the PMMA MWD for three samples of different conversions collected in the E2, E4, E6, E9, and E12 trials. The GPC analysis revealed that the samples for the E2 trial had unimodal sharp peaks and tended to shift when the monomer conversion increased from 12.1% to 34.1%. Moreover, a clear shift in the unimodal sharp peaks was also observed when analyzing the samples collected from the E4 trial, and for monomer conversion rates of 37.7, 52.6, and 64.1%. Unimodal peaks were obtained when analyzing the samples collected from the E6, E9, and E12 trials, and these peaks shifted along with increases in the MMA conversion rate. Therefore, typical features of ATRP systems were observed from the data analysis of the polymerization system [24]. 

**Figure 6:**
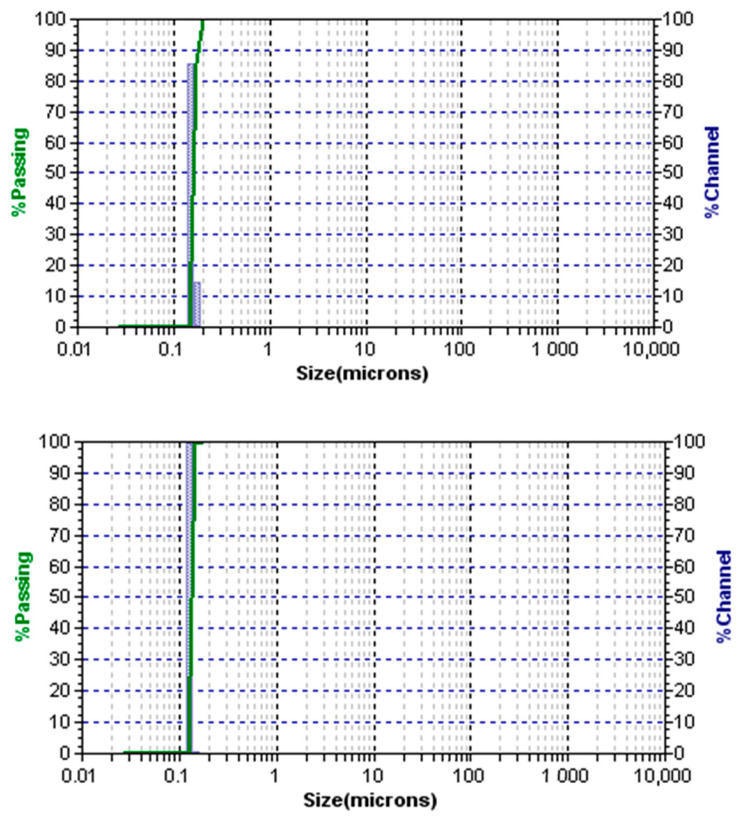
Laser diffraction (LD): image of the final polymer product of the E2 run (**Top**) and the final polymer product of the E4 run (**Bottom**). Both images for samples collected after 5 h of polymerization initiation.

The Microtrac analysis presented in Figure 6 shows an average particle size of approximately 154 nm for E2 trial and 133 nm for E4 trial after 5 h of reaction time. The E4 trial received more surfactant. Therefore, larger areas between the dispersed and continuous phases are covered by the addition of the adsorbed surfactant, which caused the particle size to diminish. 

**Figure 7:**
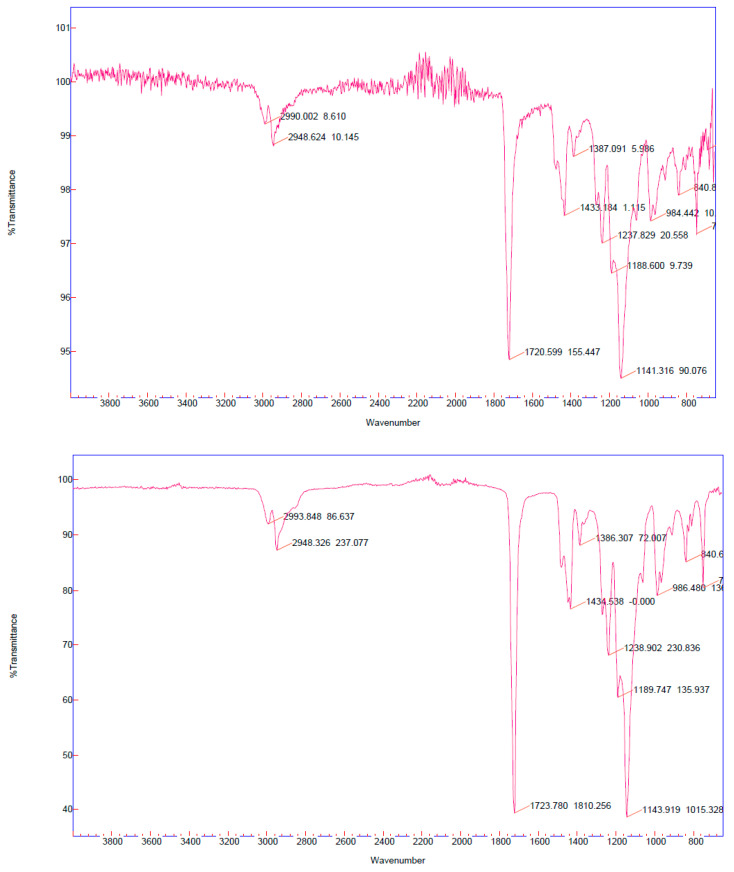
FTIR spectrometry: image of final polymer product of the E2 run (**top**) and the E4 run (**bottom**) using the 4,4′-Dinonyl-2,2′-dipyridyl (dNbpy) ligand for a sample collected after 3 h of polymerization initiation.

Figure 7 displays the FTIR spectrometry analysis of the PMMA sample collected after 3 h of polymerization for the E2 (top) and E4 (bottom) trials, respectively. There is a distinct absorption band ranging from 1140 to 1240 cm^−1^. This was attributed to the C–O–C stretching vibration. The bands at 1386.5 and 748 cm^−1^ are attributed to the α-methyl group vibrations. The band at 986 cm^−1^ is the characteristic absorption vibration of the PMMA, along with the band at 840 cm^−1^. In both cases, the band at 1721.5 cm^−1^ shows the presence of the acrylate carboxyl group. Furthermore, the band at 1434 cm^−1^ can be attributed to the bending vibration of the C–H bonds of the –CH_3_ group. The two bands at 2991 and 2948 cm^−1^ can be attributed to the C–H bond stretching vibrations of the –CH_3_ and –CH_2_– groups, respectively. Based on the above discussions, it can be concluded that the prepared polymer for all trials was indeed a macromolecular PMMA [53].

**Figure 8:**
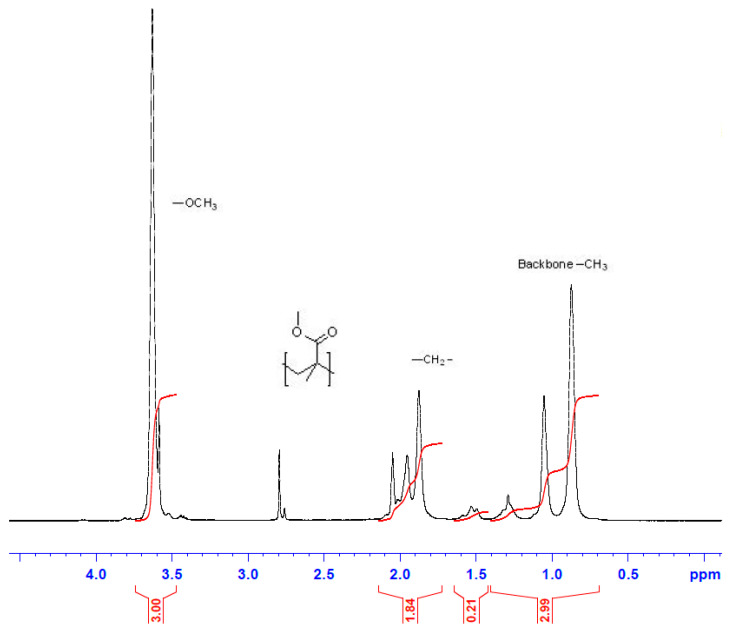
NMR spectrometry: image of final polymer product of the E2 run for a sample collected after 3 h of polymerization.

Figure 8 displays the ^1^H-NMR structure characterization for PMMA sample collected at 3 h of reaction time for E2 trial. The proton methyl group (*n-*C(CH_3_)(COOCH_3_)) was observed by the chemical shift at 0.84–1.25 ppm. The peak at 1.88–2.08 ppm was attributed to the protons of the methylene groups (–CH_2_–). The protons of the methyl ester groups (*n-*C(CH_3_)(COOCH_3_)) were assigned to the chemical shift centered at 3.65 ppm [54,55]. The protons of the methyl ester groups at the side chain were represented by the absorptions at 3.78 ppm, which is different from the methyl ester protons in the PMMA at 3.6 ppm. This occurred because of the electron-withdrawing effect of the ω-Br atom, as suggested by Ouchi et al. [56].

## 4. Conclusions

The polymerization of MMA AGET ATRP was successfully carried out using a two-step emulsion procedure in a 2 L stirred batch reactor. The colloidal stability is a dominant challenge for emulsion AGET ATRP and requires further investigation. The occurrence of polymer coagulation was identified as a detrimental issue when the temperature was equal to or above 60 °C. However, the results demonstrated that the polymer coagulation could be avoided while high monomer conversion is still possible at mild reaction temperature. The results also showed that more surfactant added to the system with higher stirring speed has stabilized the emulsion AGET ATRP of MMA. For 5 h of polymerization at a reaction temperature of 50 °C, the MMA conversion rate reached 64.1% to produce a PMMA with an average molecular weight of 4.5 kg/mol and a Ð of 1.17. In contrast, adding more water has led to a loss of control over the system since the monomer and catalyst complex undergo a phase partitioning. In conclusion, high conversion rates and good latex stability are possible to attain by selecting suitable reaction recipients in the two-step AGET ATRP emulsion polymerization. Finally, more extensive studies about the surfactant types and amount are recommended since the nature of AGET ATRP polymerization system is highly dominated by micelles nucleation.

## Figures and Tables

**Table 1:** Experimental conditions of the emulsion activators generated by electron transfer (AGET) atom transfer radical polymerization (ATRP) of methyl methacrylate (MMA) using the 4,4′-Dinonyl-2,2′-dipyridyl (dNbpy) ligand.

Exp.	T	Mixing Speed	EBiB	Brij 98	dNbpy	Ascorbic Acid	CuBr_2_	Water
(-)	(°C)	(rpm)	(g)	(g)	(g)	(g)	(g)	(mL)
E1	60	250	0.4981	15.0064	0.3065	0.0608	0.0837	350
E2	50	250	0.4988	15.0043	0.3061	0.0611	0.0839	350
E3	60	250	0.6140	22.3820	0.2921	0.1425	0.0802	350
E4	50	250	0.6147	22.3807	0.2923	0.1422	0.0809	350
E5	40	250	0.6138	22.3832	0.2918	0.1420	0.0805	350
E6	50	250	0.6144	05.0022	0.2921	0.1423	0.0807	350
E7	50	250	0.6139	10.0037	0.2923	0.1419	0.0804	350
E8	50	250	0.6141	15.0016	0.2918	0.1422	0.0803	350
E9	50	250	0.6136	22.3845	0.2921	0.1421	0.0805	400
E10	50	250	0.6140	22.3811	0.2923	0.1421	0.0801	450
E11	50	250	0.6149	22.3806	0.2918	0.1420	0.0809	500
E12	50	100	0.6140	22.3831	0.2921	0.1420	0.0800	350
E13	50	150	0.6132	22.3807	0.2923	0.1419	0.0806	350

**Table 2:** Experimental results of the emulsion AGET ATRP of MMA using the dNbpy ligand.

Time (h)	1 h			3 h			5 h		
Exp.	Conv	M_n_	Ð	Conv	M_n_	Ð	Conv	M_n_	Ð
(-)	(%)	(g/mol)	(-)	(%)	(g/mol)	(-)	(%)	(g/mol)	(-)
E1	24.7	7084	1.35	51.5	14,396	1.30	–	–	–
E2	12.1	2719	1.29	24.9	3913	1.26	34.1	5340	1.15
E3	47.3	4877	1.53	68.7	5365	1.40	–	–	–
E4	37.7	1828	1.19	52.6	3318	1.18	64.1	4596	1.17
E5	27.1	2463	1.35	41.9	2888	1.32	51.7	3416	1.27
E6	19.7	2148	1.34	34.7	4794	1.35	42.3	5243	1.36
E7	26.9	6449	1.34	42.6	6735	1.35	56.6	7172	1.32
E8	28.1	5290	1.27	48.3	6636	1.23	59.2	7423	1.22
E9	35.9	3412	1.17	50.7	5276	1.18	60.0	6077	1.15
E10	21.8	3175	1.22	40.6	4758	1.14	49.3	5221	1.23
E11	17.9	5105	1.27	32.0	6749	1.53	42.5	8415	1.49
E12	28.2	2612	1.14	42.6	5239	1.15	59.3	7860	1.13
E13	30.6	3338	1.16	47.2	4371	1.18	61.2	5169	1.15
